# Clinical Significance of SASH1 Expression in Glioma

**DOI:** 10.1155/2015/383046

**Published:** 2015-09-06

**Authors:** Liu Yang, Haitao Zhang, Qi Yao, Yingying Yan, Ronghua Wu, Mei Liu

**Affiliations:** ^1^Department of Neurosurgery, Affiliated Hospital of Nantong University, Nantong 226001, China; ^2^Key Laboratory of Neuroregeneration, Nantong University, Nantong 226001, China

## Abstract

*Objective.* SAM and SH3 domain containing 1 (*SASH1*) is a recently discovered tumor suppressor gene. The role of SASH1 in glioma has not yet been described. We investigated SASH1 expression in glioma cases to determine its clinical significance on glioma pathogenesis and prognosis. *Methods.* We produced tissue microarrays using 121 patient-derived glioma samples and 30 patient-derived nontumor cerebral samples. Immunohistochemistry and Western blotting were used to evaluate SASH1 expression. We used *Fisher*'s exact tests to determine relationships between SASH1 expression and clinicopathological characteristics; Cox regression analysis to evaluate the independency of different SASH1 expression; Kaplan-Meier analysis to determine any correlation of SASH1 expression with survival rate. *Results.* SASH1 expression was closely correlated with the WHO glioma grade. Of the 121 cases, 66.9% with low SASH1 expression were mostly grade III-IV cases, whereas 33.1% with high SASH1 expression were mostly grades I-II. Kaplan-Meier analysis revealed a significant positive correlation between SASH1 expression and postoperative survival. *Conclusions.* SASH1 was widely expressed in normal and low-grade glioma tissues. SASH1 expression strongly correlated with glioma grades, showing higher expression at a lower grade, which decreased significantly as grade increased. Furthermore, SASH1 expression was positively correlated with better postoperative survival in patients with glioma.

## 1. Introduction

The annual incidence rate of brain glioma (glioma for short) has increased, making it the most common malignant intracranial tumor (~50% of cases) [1]. High-grade gliomas have no capsule or clear boundaries with surrounding normal tissue and typically show invasive growth. Because of these characteristics, the total resection rate of glioma is low, radiotherapy is often contraindicated, and chemotherapy is limited because of the impenetrability of the blood brain barrier; therefore, treatment strategies are largely ineffective, leading to high relapse rates. The 5-year survival rate for patients with glioma is poor (20–30%). Of those with poor 5-year survival rates, 50% could be accounted for by those with highly malignant tumors who survived for <1 year [2]. Therefore, there is an urgent need to discover new druggable targets to develop novel glioma treatments that might enhance patient survival.

With the development of molecular biological techniques, identifying novel high-specification therapeutic targets has become a new direction for glioma research. There has been an emphasis on scaffold proteins, which play an important role in the regulation of signal transduction. Scaffold proteins are biologically inert but bind to their respective substrates through the action of auxiliary phosphatases and protein kinases, thus exerting inflammatory response effects. They also mediate vascular endothelial cell contraction and strengthen phagocytic cells [3]. In malignant tumors, the downregulation and deactivation of scaffold proteins cause aberrant signaling [4].

SASH1 (SAM and SH3 domain containing 1) is a newly discovered scaffold protein, which is considered as a tumor inhibitor. In 2003, Zeller et al. [5] discovered a loss of* SASH1* heterozygosity in chromosome 6q24.3, a location where many factors are considered to harbor tumor suppressor function. SASH1 expression was significantly deceased or absent in various cancers including colorectal cancer [6], melanoma [7], osteosarcoma [8], and lung cancer [9].

SASH1, together with related molecules, regulates cytoskeletal proteins and promotes cell and matrix adhesion [10, 11]. In addition, Zhou et al. found that SASH1 affected E-cadherin signaling to regulate transepithelial migration [12]. However, the specific mechanism of how SASH1 affects the biological behavior of a tumor is unclear, and the impact of SASH1 expression on glioma is yet to be determined. We previously studied the impact of SASH1 on the biological behavior of glioma cells and found that after overexpressing SASH1 plasmid U251 glioma cells exhibited significantly reduced cell viability, proliferation, and invasion and a significantly higher apoptotic index [13]. We then suggested SASH1 gene may play a tumor inhibitory role in glioma cells. Therefore in this present study, we analyzed SASH1 protein expression in patient-derived glioma and nontumorous tissues to evaluate possible associations of SASH1 expression with clinicopathological characteristics (age, sex, and tumor grade) and patient prognosis, to provide some clinical data for our further study.

## 2. Material and Methods

### 2.1. Glioma Patient Specimens

We collected 121 patient-derived paraffin-embedded glioma tissues from the Department of Pathology, the Affiliated Hospital of Nantong University, between 2005 and 2013. Patients with autoimmune diseases or recurrent glioma were excluded. Patients did not undergo any other treatments before surgery. The mean patient age was 50.3 years (range, 6–86 years), 79 (65.3%) were male, and 42 (34.7%) were female. Follow-up data were completed for all patients, with a median follow-up time of 31 months (range, 1–116 months). The postoperative diagnosis was confirmed histologically. All cases were reevaluated for grade and histological type by two independent pathologists. According to the World Health Organization (WHO) 2007 pathological classification standards concerning central nervous system tumors, patients with glioma were subdivided as having low-grade glioma (grade I, 7 cases; grade II, 31 cases) or high-grade glioma (grade III, 38 cases; grade IV, 45 cases). As a control, we included 30 patient-derived paraffin-embedded tissues from patients with brain contusion (nontumorous tissue). This study was approved by the Ethics Committee of the Affiliated Hospital of Nantong University, and informed consent was obtained from each patient or their proxy.

### 2.2. Tissue Microarray (TMA) Construction and Immunohistochemistry (IHC) Analysis

As described in previous studies [14, 15], the 121 glioma and 30 nontumor tissues were used to construct TMAs. A representative area of each sample was selected and 2.0 mm tissue cores were designed to construct a tissue microarray system (Quick-Ray, UT06, UNITMA, Korea) in the Department of Clinical Pathology, Nantong University. We used hematoxylin-eosin staining (H&E) to confirm the quality of TMA sections. IHC staining was performed as described previously [16], and deparaffinized sections from array blocks were separately stained using an Autostainer Universal Staining System (LabVision, Kalamazoo, MI, USA). Briefly, sections (4 *μ*m) were deparaffinized and rehydrated. Antigen retrieval was performed by boiling under pressure in 0.01 M citrate buffer, pH 6.0, for 3 min. Nonspecific binding was blocked by incubation in 5% goat serum in phosphate-buffered saline (PBS) for 15 min, and the tissues were incubated with polyclonal rabbit anti-SASH1 antibody (1 : 100, Santa Cruz Biotechnology Inc., CA, USA) overnight. The secondary antibody used was horseradish peroxidase-conjugated anti-rabbit antibody (DakoCytomation, Carpinteria, CA, USA). For negative controls, PBS was used instead of the primary antibody. Blind SASH1 immunostaining evaluation and independent observation were simultaneously performed. IHC results were analyzed according to a previously described method [14].

### 2.3. Interpretation of Immunostaining Results

SASH1 expression was scored in terms of the proportion of positive cells and the staining intensity. The percentage of SASH1-positive cells was scored into four categories, with a score of 0 given for <10%, 1 for 10–40%, 2 for 41–60%, and 3 for >61%. Staining intensity was scored as follows: 0 (negative), 1 (weakly positive), 2 (moderately positive), and 3 (strongly positive). The product of the intensity and percentage scores was used as the final SASH1 staining score. The cut-off point for the SASH1 expression score that was statistically significant in terms of survival was set using the X-tile software program (The Rimm Lab at Yale University) as described previously [14]. A total score <4 was defined as low expression, and that of ≥4 was defined as high expression.

### 2.4. Protein Extraction and Western Blot Analysis

From the Affiliated Hospital of Nantong University, we collected 9 fresh brain tissue contusion samples and 12 fresh glioma tissue samples. Harvested tissues were stored in liquid nitrogen until their use in western blot analysis. These cases were reevaluated for grade and histological type by the same two pathologists above.

Western blotting was performed according to standard protocols as described in our previous study [17]. Tissues were cut into small pieces and homogenized in a glass homogenizer in extraction buffer provided in the protein extraction kit (Bio-Rad, Richmond, CA, USA) including 1% protease inhibitors (Bio-Rad) and lysed on ice. After centrifugation, the protein concentration of the supernatant was analyzed using a bicinchoninic acid assay kit (Bio-Rad, Richmond, CA). Forty *μ*g of total protein from each sample was separated by SDS-PAG Electrophoresis and transferred to a polyvinylidene difluoride membrane (Millipore Company, Bedford, MA). Western blotting was performed according to standard protocols using the following antibodies: goat anti-glyceraldehyde 3-phosphate dehydrogenase (GAPDH) polyclonal (1 : 800; Santa Cruz Biotechnology Inc.), rabbit anti-SASH1 polyclonal (1 : 500; Santa Cruz) donkey anti-goat IRDye (1 : 10,000; Rockland, Limerick, PA, USA), and donkey anti-rabbit IRDye (1 : 10,000; Rockland). Immunoblots were analyzed using the Odyssey densitometry program (LI-COR, Lincoln, NE, USA). GAPDH was used as a loading control.

### 2.5. Statistical Analyses

*Fisher*'s exact test, Cox regression analyses, and *t*-tests were used to analyze the expression pattern of SASH1 and its association with clinicopathological characteristics and postoperative survival. The Kaplan-Meier method was used to generate survival curves. All data were processed using SPSS20, and a *P* value of <0.05 was considered as statistically significant.

## 3. Results

### 3.1. Expression of SASH1 Protein in Glioma and Nontumor Brain Tissue

**Figure 1:**
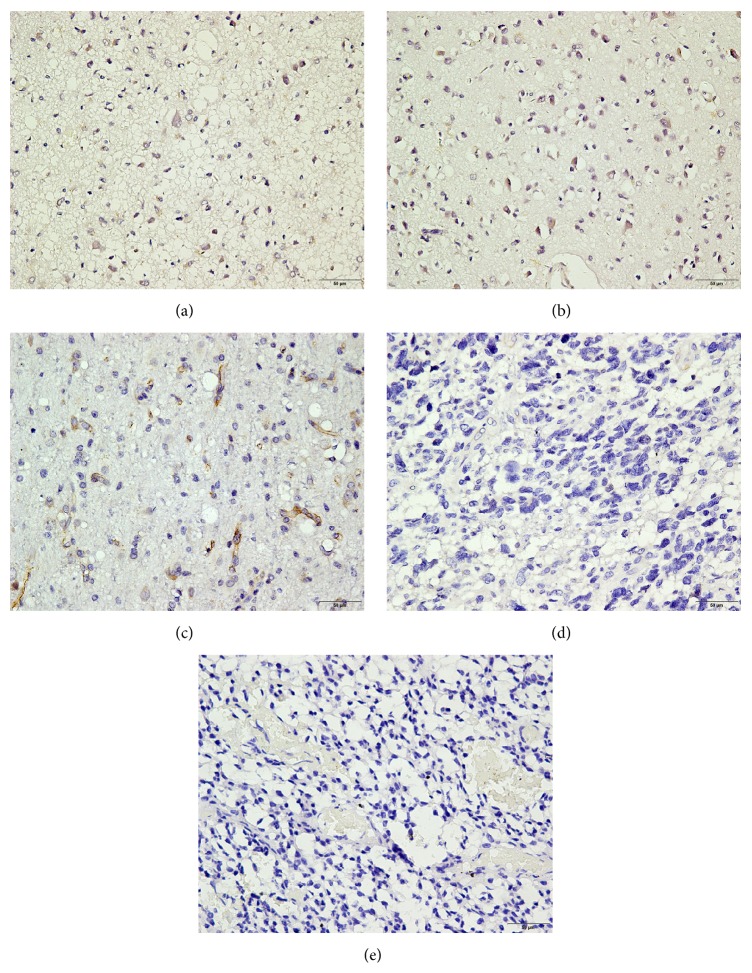
Immunohistochemical detection of SASH1 expression in nontumor and glioma tissues. (a) Nontumorous tissue, (b) grade I glioma, (c) grade II glioma, (d) grade III glioma, and (e) grade IV glioma. SASH1, in brown; nuclei, in blue. Bar = 50 *μ*m.

To investigate the expression of SASH1 protein in glioma and nontumorous tissue, we performed IHC analysis on primary patient-derived tissues. The typically observed SASH1 staining patterns are shown in Figure 1(a). Brown staining can be seen, primarily in cytoplasm of cells. SASH1 expression levels were highest in nontumorous tissues (Figure 1(a)) which were observed from all the 30 cases of control samples. In glioma tissues, as tumor grade increased, SASH1 expression decreased (Figures 1(b) and 1(c)) or was completely absent (Figures 1(d) and 1(e)). Of the 121 cases of glioma, 66.9% (81 cases) had low SASH1 expression and these were mostly grade III-IV cases (26 + 40), whereas 33.1% (40 cases) had high SASH1 expression and these were mostly grades I-II (4 + 19).

**Figure 2:**
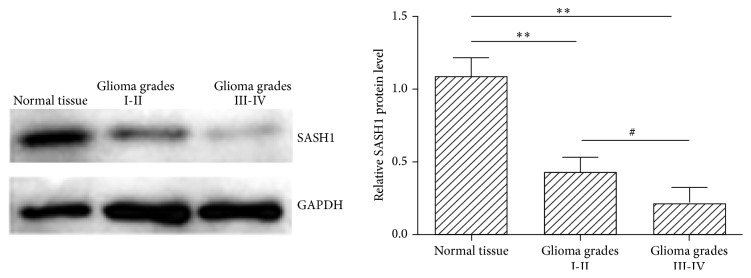
Western blotting of SASH1 protein level in glioma and normal tissues. The left panel is a representative result of western blotting. SASH1 protein expression was calculated by normalizing SASH1 intensity to GAPDH intensity, and data was compared to the normal tissue, represented as 1. Data are expressed as mean ± SEM; ^*∗∗*^ *P* < 0.01 versus normal tissue; ^#^ *P* < 0.05 between different grades.

Western blotting revealed that SASH1 expression in glioma tissues was significantly lower than that in nontumor tissues (Figure 2). This observation was significantly more apparent in high-grade gliomas (III-IV), which showed the lowest protein level with a 74% decrease compared with controls, while in low-grade gliomas (I-II) SASH1 expression was decreased by 60% compared with controls. The differences in SASH1 expression levels between nontumor tissues, low-grade glioma, and high-grade glioma were all statistically significant.

### 3.2. Correlation of SASH1 Expression with Clinicopathological Parameter of Glioma

SASH1 expression and the clinicopathological characteristics in glioma patients are shown in Table 1. Statistical analysis revealed that SASH1 expression was not correlated with age or sex (*P* > 0.05) but that SASH1 expression was significantly correlated with glioma grade, with higher expression at lower grades, and vice versa (*P* < 0.01).

### 3.3. Relationship between SASH1 Expression and Prognosis in Patients with Glioma

**Figure 3:**
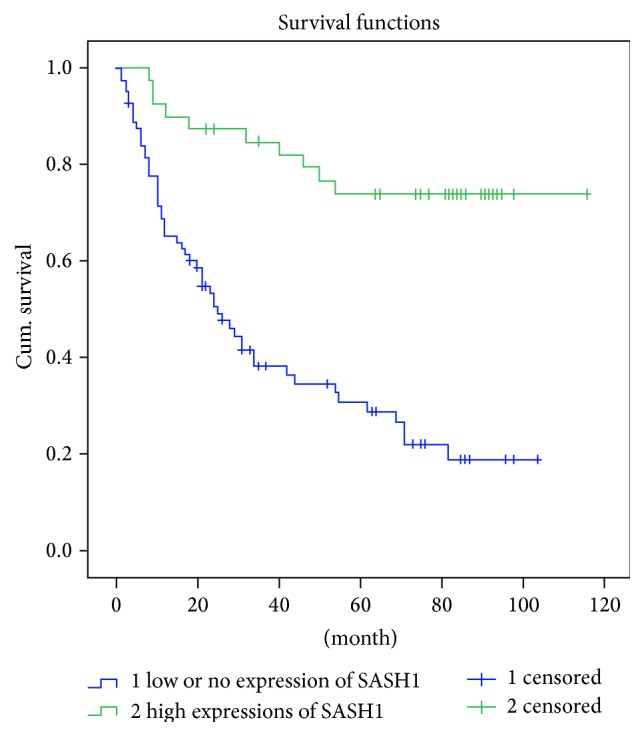
Kaplan-Meier survival curves following surgical therapy in glioma. Patients with low or no expression of SASH1 in glioma (blue line) exhibited significantly poorer survival compared with the high expression group (green line).

Till December 31, 2014, 55 patients of all 121 cases were alive, and the average of survival time was 65.9 months; 66 patients were dead, and the average of survival time was 22.4 months. And the average of survival time of total grouped 121 cases was 42.1 months. According to Kaplan-Meier analysis, there was a significant positive correlation between SASH1 expression and postoperative survival in patients with glioma (*P* < 0.05). The higher the SASH1 expression levels, the longer the postoperative survival, and vice versa (Table 2 and Figure 3). Similarly, sex and glioma grades and SASH1 expression were significantly associated with survival status that employed the Cox regression model (Table 3). According to the results of this regression analysis, low expression of SASH1 is an independent prognostic factor for shorter survival in patients with glioma.

## 4. Discussion

In the present study, immunohistochemical and western blot analyses of SASH1 protein expression revealed that SASH1 expression was much higher in nontumorous tissues compared with glioma tissues, and that expression was closely correlated with glioma grade. SASH1 expression was lower at higher grades, and vice versa. However, SASH1 expression was not dependent on patient age or sex. In addition, Kaplan-Meier survival analysis revealed that patients with higher SASH1 expression had much better postoperative survival.

Existing studies have indicated that SASH1 is a tumor suppressor [6]. The deactivation of tumor suppressor genes often leads to cell transformation and tumorigenesis, and many studies have shown that the loss of tumor suppressor gene function in regulating cell transduction, cell cycle, apoptosis, and other important biological processes [11, 18–20] could play a pivotal role in the development of glioma [10, 21, 22]. Zeller et al. indicated that SASH1 mRNA expression was decreased in 74% of their clinical breast cancer cases and that, in thyroid cancer, decreased SASH1 expression was closely correlated with tumor progression and prognosis [5]. Rimkus et al. found that SASH1 expression in colon cancer tissues decreased as cancer TNM stage increased [6]. Moreover, in the colon cancer group, compared to those without liver metastasis, those with liver metastasis had much lower SASH1 expression. Additionally, Meng et al. [8] found that SASH1 was significantly lower in human osteosarcoma MG-63 cells and that the SASH1 expression in tumor tissues with lung metastasis was significantly lower than in tumor tissues without lung metastasis. With advancing tumor stage, SASH1 mRNA expression decreased. Induction of SASH1 expression reduced cyclin D1 and matrix metalloprotease- (MMP-) 9 expression and increased caspase 3 expression, which suggested that overexpression of SASH1 in human osteosarcoma MG-63 cells might inhibit cell growth, proliferation, and invasion and promote apoptosis. Both of these studies suggested an association of decreased SASH1 expression with tumor progression and metastases. We reported in previous study that cyclin D1 and MMP-9 expression were decreased, and caspase 3 expression was increased by overexpression of SASH1 in U251 cells [13], supporting the role of SASH1 as a candidate tumor suppressor. Similar effects were observed in human lung adenocarcinoma A549 cells [9] and human melanoma A-375 cells [7].

Similar to these previously published observations our results suggest a putative role for SASH1 in the genesis of glioma and may indicate that SASH1 gene deletions might occur to varying degrees during tumor progression. Therefore, we hypothesize that SASH1 expression level could be closely and negatively correlated with glioma grade. Current literature indicates that the SLY family, to which SASH1 belongs, has two important domains, namely, a SAM domain and a SH3 domain. The SLY family member SAMSN1 plays a role in the activation of B cells [23], whereas SASH3 is involved in regulating the adaptive immune system [24]. SASH1 is not highly expressed in the immune system, and its molecular weight (140 kDa) is much larger than that of the same family members. In addition to the SLY domain, there is an additional SAM motif at the C-terminus, and SASH1 has proline-rich regions. These features are characteristic of intracellular signaling molecules, linker molecules, and scaffold proteins, which play an important role in protein-protein interactions [25–27]. However, as a tumor suppressor gene, the roles of SASH1 in normal astrocytes on controlling cell signaling require further investigation. Recently, we performed immunoprecipitation (IP) using SASH1 antibody combined with mass spectrometry (MS) to obtain some SASH1 interacting proteins in cultured astrocytes and in an attempt to uncover molecular mechanism for SASH1 function.

## 5. Conclusion

SASH1 expression was significantly correlated with glioma grade, showing decreased expression at more advanced stages. Furthermore, higher SASH1 expression was associated with better postoperative survival in patients with glioma. These data showed that SASH1 could be a novel prognostic and therapeutic target for human glioma.

## Figures and Tables

**Table 1:** Correlations of SASH1 expression with clinicopathological characteristics in patients with glioma.

Clinicopathological parameters	Number	SASH1 expression		*P* value^a^
		Low (including negative)	High	
Total	121	81	40	
Sex				1.000
Male	79	53	26
Female	42	28	14
Age (years)				0.176
≤50	58	35	23
>50	63	46	17
WHO glioma grade				<0.0001
I	7	3	4	
II	31	12	19	
III	38	26	12	
IV	45	40	5	

^a^Statistical analyses were conducted using *Fisher*'s exact probability. *P* < 0.05 was considered as statistically significant.

**Table 2:** Survival status and clinicopathological parameters in 121 human glioma specimens.

Clinicopathological parameters	Number	Survival status		*P* value^a^
		Alive	Dead	
Sex				0.179
Male	79	32	47	
Female	42	23	19	
Age (years)				0.365
≤50	58	29	29	
>50	63	26	37	
WHO glioma grade				0.00017
I	7	7	0	
II	31	20	11	
III	38	13	25	
IV	45	15	30	
SASH1 expression				<0.0001
Low	81	25	56	
High	40	30	10	
WHO classification				
Choroid plexus papilloma	2			
Pilocytic astrocytoma	4			
Subependymoma	1			
Fibrillary astrocytoma	9			
Protoplasmic astrocytoma	6			
Gemistocytic astrocytoma	5			
Oligodendroglioma	4			
Ependymoma	3			
Oligoastrocytoma	4			
Anaplastic astrocytoma	29			
Anaplastic oligoastrocytoma	4			
Anaplastic oligodendroglioma	5			
Glioblastoma	43			
Gliosarcoma	2			

^a^Statistical analyses were performed using *Fisher*'s exact probability. *P* < 0.05 was considered as statistically significant.

**Table 3:** Survival status and clinicopathological parameters in 121 human glioma specimens by multivariate Cox regression analyses.

Parameters	Haz. ratio	Std. Err.	*z*	*P* value^a^	[95% Conf. interval]	
Age (≤50; >50)	1.0067	0.0066	1.02	0.309	0.9938216	1.019795
Sex (male; female)	0.5929	0.1298	−2.39	0.017^*∗*^	0.3860409	0.9105162
Grade (I-II; III-IV)	1.8495	0.4351	2.61	0.009^*∗*^	1.166297	2.932766
SASH1 (low; high)	0.9938	0.0014	−4.49	0.000^*∗*^	0.9911733	0.9965267

^a^Statistical analyses were performed using Cox regression analysis. ^*∗*^ *P* < 0.05 was considered as statistically significant.

## References

[1] Sciumè G., Santoni A., Bernardini G. (2010). Chemokines and glioma: invasion and more. *Journal of Neuroimmunology*.

[2] Sayegh E. T., Kaur G., Bloch O., Parsa A. T. (2014). Systematic review of protein biomarkers of invasive behavior in glioblastoma. *Molecular Neurobiology*.

[3] Chan C., Liu X., Wang L., Bardwell L., Nie Q., Enciso G. (2012). Protein scaffolds can enhance the bistability of multisite phosphorylation systems. *PLoS Computational Biology*.

[4] Chodniewicz D., Klemke R. L. (2004). Regulation of integrin-mediated cellular responses through assembly of a CAS/Crk scaffold. *Biochimica et Biophysica Acta—Molecular Cell Research*.

[5] Zeller C., Hinzmann B., Seitz S. (2003). SASH1: a candidate tumor suppressor gene on chromosome 6q24.3 is downregulated in breast cancer. *Oncogene*.

[6] Rimkus C., Martini M., Friederichs J. (2006). Prognostic significance of downregulated expression of the candidate tumour suppressor gene SASH1 in colon cancer. *British Journal of Cancer*.

[7] Lin S., Zhang J., Xu J. (2012). Effects of SASH1 on melanoma cell proliferation and apoptosis in vitro. *Molecular Medicine Reports*.

[8] Meng Q., Zheng M., Liu H. (2013). SASH1 regulates proliferation, apoptosis, and invasion of osteosarcoma cell. *Molecular and Cellular Biochemistry*.

[9] Chen E.-G., Chen Y., Dong L.-L., Zhang J.-S. (2012). Effects of SASH1 on lung cancer cell proliferation, apoptosis, and invasion in vitro. *Tumor Biology*.

[10] Alexiou G. A., Voulgaris S. (2010). The role of the PTEN gene in malignant gliomas. *Neurologia i Neurochirurgia Polska*.

[11] Marcel V., Dichtel-Danjoy M.-L., Sagne C. (2011). Biological functions of p53 isoforms through evolution: lessons from animal and cellular models. *Cell Death and Differentiation*.

[12] Zhou D., Wei Z., Deng S. (2013). SASH1 regulates melanocyte transepithelial migration through a novel Galphas-SASH1-IQGAP1-E-Cadherin dependent pathway. *Cellular Signalling*.

[13] Yang L., Liu M., Gu Z., Chen J., Yan Y., Li J. (2012). Overexpression of SASH1 related to the decreased invasion ability of human glioma U251 cells. *Tumour Biology*.

[14] Huang M., Zhu H., Feng J., Ni S., Huang J. (2015). High CD133 expression in the nucleus and cytoplasm predicts poor prognosis in non-small cell lung cancer. *Disease Markers*.

[15] Feng J., Xu L., Ni S. (2014). Involvement of FoxQ1 in NSCLC through regulating EMT and increasing chemosensitivity. *Oncotarget*.

[16] Huang J., Zhang X., Tang Q. (2011). Prognostic significance and potential therapeutic target of VEGFR2 in hepatocellular carcinoma. *Journal of Clinical Pathology*.

[17] Liu M., Wu R., Yang F. (2013). Identification of FN1BP1 as a novel cell cycle regulator through modulating G1 checkpoint in human hepatocarcinoma Hep3B cells. *PLoS ONE*.

[18] Naguib A., Cooke J. C., Happerfield L. (2011). Alterations in PTEN and PIK3CA in colorectal cancers in the EPIC Norfolk study: associations with clinicopathological and dietary factors. *BMC Cancer*.

[19] Rajan N., Elliott R., Clewes O. (2011). Dysregulated TRK signalling is a therapeutic target in CYLD defective tumours. *Oncogene*.

[20] Pustišek N., Šitum M. (2011). Uv-radiation, apoptosis and skin. *Collegium Antropologicum*.

[21] Kim Y.-J., Cho Y.-E., Kim Y.-W., Kim J.-Y., Lee S., Park J.-H. (2008). Suppression of putative tumour suppressor gene GLTSCR2 expression in human glioblastomas. *The Journal of Pathology*.

[22] Qu M., Jiao H., Zhao J. (2010). Molecular genetic and epigenetic analysis of NCX2/SLC8A2 at 19q13.3 in human gliomas. *Neuropathology and Applied Neurobiology*.

[23] Zhu Y. X., Benn S., Li Z. H. (2004). The SH3-SAM adaptor HACS1 is up-regulated in B cell activation signaling cascades. *The Journal of Experimental Medicine*.

[24] Beer S., Scheikl T., Reis B., Hüser N., Pfeffer K., Holzmann B. (2005). Impaired immune responses and prolonged allograft survival in Sly1 mutant mice. *Molecular and Cellular Biology*.

[25] Kim C. A., Bowie J. U. (2003). SAM domains: uniform structure, diversity of function. *Trends in Biochemical Sciences*.

[26] Aviv T., Lin Z., Lau S., Rendl L. M., Sicheri F., Smibert C. A. (2003). The RNA-binding SAM domain of Smaug defines a new family of post-transcriptional regulators. *Nature Structural Biology*.

[27] Mayer B. J. (2001). SH3 domains: complexity in moderation. *Journal of Cell Science*.

